# How AI will redefine care delivery: the rise of the generalist-specialist

**DOI:** 10.1093/haschl/qxag075

**Published:** 2026-03-26

**Authors:** Bob Kocher, Siobhan Nolan-Mangini, Robert M Wachter

**Affiliations:** Stanford Department of Health Policy, Stanford School of Medicine, Stanford, CA, United States; Venrock, Palo Alto, CA, United States; Venrock, Palo Alto, CA, United States; Department of Medicine, University of California San Francisco, San Francisco, CA, United States

**Keywords:** artificial intelligence, primary care, Academic Medical Centers, care model redesign, artificial intelligence in care delivery, medical education, specialty care model, healthcare economics, healthcare policy

## Abstract

Artificial intelligence is transforming healthcare beyond efficiency gains, presenting an opportunity to fundamentally reorganize the clinical workforce. This paper proposes the “Generalist-Specialist” model, arguing that AI's capacity to scale specialist-level knowledge challenges the historical “cognitive necessity” for narrow specialty definitions. By democratizing clinical expertise, AI-augmented clinicians could manage the full constellation of patients' chronic and complex conditions within broader, disease-based domains (eg, cardiometabolic, infectious and inflammatory) rather than organ-specific specialties. This shift promises fewer handoffs, better coordination, and unlocked specialty capacity for patients who need it most. Economically, consolidating care under fewer clinicians makes value-based and bundled payments more feasible, though under fee-for-service, without countervailing payment reform, it risks increasing total utilization. Realizing this vision requires evolving medical education to incorporate AI, reforming malpractice standards to accept AI-guided evidence-based care, modernizing credentialing frameworks, and strategically repositioning Academic Medical Centers toward ultra-complex care or seamless generalist-specialist hubs. The greatest impact of AI in healthcare may not be doing the same things more efficiently, but enabling an entirely new class of clinicians organized around disease biology and patient need. Realizing this potential will require navigating formidable non-technical barriers, including incumbent interests, legacy payment models, and patient safety and liability standards.

Artificial intelligence is rapidly transforming healthcare, with most doctors now using ambient scribes and AI-based billing and clinical decision support.^1,2^ These developments, while significant, largely offer efficiency gains within the existing system. One area that has been less explored is how ubiquitous access to AI will blur and redefine how clinicians train, organize, and practice. In particular, AI's ability to scale specialist-level knowledge across the workforce may prove transformative.

## The declining relevance of traditional specialty care definitions

Today's medical workforce is organized into a rigid hierarchy of specialties. Each specialty possesses its own governing body, board exams, and scope of practice. Historically, this narrowly defined scope was shaped by cognitive necessity: the corpus of medical knowledge was simply too vast for any single person to master and remain current on. The development of defined specialties was codified by specialty-specific training programs, specialty societies, billing rules, and standards of care that often-determined liability.

The result for patients, however, is a fragmented experience that requires many doctors and is plagued by challenges in care coordination. Patients with complex multimorbidities are often shuffled between cardiologists, nephrologists, and endocrinologists, each treating an organ system with limited visibility into the whole.

The “cognitive necessity” argument is challenged by generative AI. Today's leading AI systems excel at knowledge-retrieval tasks: they pass Board exams across multiple specialties^3^ and generate recommendations consistent with specialist guidelines. What current models do less reliably is context-dependent, longitudinal clinical reasoning—synthesizing fragmented histories, eliciting subtle findings, and navigating patient-specific uncertainty over time. These are hard problems, but the pace of improvement is rapid. In our unpublished experiments, physician and AI specialty referrals showed remarkable concordance, with similarly precise management instructions.

If AI models can make the expertise of traditional specialties available to generalists, this raises a fundamental question: Why do we need a care model defined narrowly by specialty?

## A way to organize care delivery: the “generalist-specialist” model

The democratization of specialist-level clinical knowledge invites us to reimagine the medical workforce. Physician care could be generalized into two broad categories: those who diagnose, prescribe, and manage patients over time, and those who perform physical interventions such as surgery. While this split might be too reductive, a reorganization around broad disease areas rather than organ-specific specialties offers a pragmatic middle ground. Care could be consolidated into groupings such as:

- Cardiometabolic diseases: Combining cardiology, endocrinology, and nephrology.
- Infectious and Inflammatory Diseases: Merging rheumatology, infectious disease, and gastroenterology.
- Mental and Neurological Health: Unifying neurology and psychiatry.
- Oncology: A comprehensive cancer care domain.
- Interventional Care: Grouping surgery, dermatology, ophthalmology, and anesthesiology.
- Primary Care: Spanning OB/GYN, internal medicine, and pediatrics.
- Shared Services: Radiology, emergency medicine, pathology, intensive care and hospital medicine.

In this model, a single clinician, augmented by AI, would manage the entire constellation of a patient's diseases. A patient with diabetes, hypertension, and early-stage CKD would not need three specialists; they would see one cardiometabolic specialist. Similarly, a patient with Crohn's disease who develops inflammatory arthritis could be managed within a unified practice, rather than toggling between gastroenterology, rheumatology, and dermatology.

This model applies most naturally to cognitive specialties where the clinician's value lies primarily in diagnostic reasoning and longitudinal treatment management. For specialties with substantial procedural components, the implications differ. In fields like dermatology, gastroenterology, or ophthalmology (where clinicians perform both cognitive evaluation and procedures), AI is likely to shift the practice mix rather than transform the specialty. Generalist-specialists may absorb more evaluation and management work, while proceduralists concentrate on interventions. Surgical and highly interventional specialties will remain largely unaffected by this new care model.

The benefits are substantial. Patients would experience fewer handoffs, faster diagnoses, less administrative friction, and lower co-payments. And if more patients received the bulk of their care from generalist-specialists, this would unlock massive specialty care capacity, thus improving access.

We do not envision the generalist-specialist as a replacement for all subspecialty expertise. Rather, we propose a new category of AI-augmented clinicians trained across broad disease domains who manage moderate-to-high complexity patients longitudinally, reducing the volume of referrals to subspecialists while reserving those subspecialists for procedurally intensive cases, rare diagnoses, and high outliers in terms of disease acuity.

## Economic considerations

Economically, this shift would also make non-fee-for-service payment models more feasible. A major difficulty with value-based payment and bundled payments for specialty care often lies in the sheer number of clinicians involved in a single episode.

However, this model carries real economic risk under fee-for-service payment if implemented without constraint. Today, “scope of service” is defined narrowly by specialty training, hospital privileging, payer expectations, referral culture, and malpractice risk. These forces create friction that limits how much care any one clinician delivers directly. While a primary care physician may technically be able to manage more complex inflammatory diseases, they will often refer to rheumatology. This friction acts as a soft utilization control.

However, if AI expands the effective scope of generalist clinicians, that friction should decline. This new construct would not only shift volume from specialists to generalists, but it could also increase total service delivery by converting previously deferred, fragmented, or incomplete care into billable activity. Domain-based bundled and longitudinal payment models will need to evolve alongside this clinical reorganization, as, without payment reform, the result could be increased total service volume.

Specific payment experiments worth piloting include: per-member-per-month capitation tied to defined disease domains (eg, a cardiometabolic capitation covering all cardiology, endocrinology, and nephrology services for an enrolled population); global ambulatory codes that bundle cognitive evaluation and management across related conditions; and shared-savings arrangements that reward reductions in total cost of care. These approaches would align financial incentives with the expanded scope of practice of Generalist-Specialists.

## Implications for academic medical centers (AMCs) and health systems

This reorganization poses a challenge and an opportunity for Academic Medical Centers. Historically, AMCs have relied on a “hub-and-spoke” model where high-margin, complex specialty cases are funneled to them because they offer specialized expertise.

If AI allows community and generalist clinicians to manage complex conditions without referral, the volume of routine specialty care at AMCs will decline. AMCs and health systems could respond in two major ways^4^:

- Focus on the “Ultra-Complex” Tier: Cases that truly exceed AI capabilities—the “n-of-1” cases requiring research-grade intervention and complex procedures
- Reorganize as Generalist-Specialists: Build a more seamless one-stop shop for diagnostics, procedures, labs and pharmacy than community hospital systems.

## Implications for the clinical workforce

The clinical workforce will experience a transition period as specialists reassess their roles. Some will upskill further into procedural or ultra-complex care, while others will choose to adopt a broader “generalist-specialist” model supported by AI. (It should be noted that, in some U.S. markets, there is already a tradition of some medical subspecialists providing primary care.) This transition is likely to be uneven across markets and institutions, but over time, it offers a path to a more flexible, resilient clinical workforce aligned with patient needs rather than legacy specialty boundaries. This creates training opportunities, such as cross-specialty “domain certificates” (eg, cardiometabolic) and AI-era maintenance-of-certification pathways focused on outcomes and competency rather than time-in-seat.

One risk here is assuming that these specialists will be comfortable serving as quasi-PCPs. A more feasible model is that the generalist-specialist will assume a middle layer between primary care frontlines and subspecialists in highly specialized settings. In this model, we could have clinicians trained (or re-trained) to own a domain longitudinally for moderate-to-high complexity patients, with subspecialists reserved for the far end of acuity.

Patient safety is an important concern. An AI-augmented generalist-specialist managing conditions at the edges of their traditional training introduces risks that differ from those in conventional specialty care, including AI hallucinations, missed contextual signals, and errors of overconfidence. Implementation will benefit from clear escalation protocols to subspecialists and systematic human (and perhaps AI-based) oversight.

## The path forward

Realizing this vision requires specific changes, though perhaps fewer than one might expect. Medical education must pivot to training clinicians to incorporate AI into care, effectively expanding their scope of practice. Medical malpractice standards must evolve to accept AI oversight and evidence adherence, replacing reliance on defensive referrals and fellowship training or specialty board certification as the gold standard. Regulators will need to modernize privileging frameworks to allow for competency-based expansion across disease domains. Notably, new payment codes may not be immediately needed since any clinician can already bill any specialty care code if they perform the service. Over time, policies will be needed to incorporate domain-based bundled and longitudinal payments in areas such as metabolic and inflammatory disease. Patients will need to be educated that seeing fewer specialists may result in better, more coordinated care.

Specialty societies may resist initially. But a benefit of this approach is that it mitigates specialty workforce shortages and increases the proportion of clinical work on the most complex and potentially rewarding work.

The “narrow scope” era of medicine was in part necessitated by the limits of a clinician's cognitive capacity. AI has the capacity to remove this limitation. By reorganizing care around a patient's needs, we can achieve better outcomes, better access, and hopefully lower total cost of care. The biggest impact of the AI healthcare revolution may not just be doing the same things more efficiently, but instead reorganizing care around disease biology and patient need through the development of an entirely new class of generalist-specialist clinicians.

## Supplementary Material

qxag075_Supplementary_Data
